# Occupational accident and disease claims, work-related stress and job satisfaction of physiotherapists

**DOI:** 10.1186/s12995-014-0036-3

**Published:** 2014-12-02

**Authors:** Birte Brattig, Anja Schablon, Albert Nienhaus, Claudia Peters

**Affiliations:** University Medical Center Hamburg-Eppendorf (UKE), Institute for Health Services Research in Dermatology and Nursing (CVcare), Hamburg, Germany; Institute for Statutory Accident Insurance and Prevention in the Health and Welfare Services (BGW), Department of Occupational Health Research, Hamburg, Germany; University Medical Center Hamburg-Eppendorf, Institute for Health Service Research in Dermatology and Nursing, Martinistrasse 52, 20246 Hamburg, Germany

**Keywords:** Working conditions, Physiotherapist, Job satisfaction, Accidents, Occupational disease

## Abstract

**Introduction:**

Physiotherapists are exposed to diverse occupational demands. Until now, little has been known about the interaction between occupational stress and the job satisfaction of physiotherapists. This paper aims to examine their work-related stress and job satisfaction. It will analyse accidents at work and occupational diseases of physiotherapists along with work-related physical and psychosocial stress and job satisfaction.

**Method:**

We analysed routine data of the German Institute for Statutory Accident Insurance and Prevention in the Health and Welfare Services (BGW) on accidents at work and occurring en route to/from work as well as occupational diseases of physiotherapists. Work-related stress and job satisfaction were examined in a cross-sectional survey using a standard questionnaire to be completed by subjects themselves.

**Results:**

Between 2007 and 2011, 1,229 cases of occupational disease were reported to the BGW. The majority of reports involved skin diseases (73%). Stumbles and falls were the most frequent causes of accidents at work (42.9%). Eighty-five physiotherapists all over Germany took part in the survey. They experience high quantitative demands at work. The main physical demands consist of a torso posture between 45° and 90° and high hand activity. Of the 85 subjects, 51% suffer from complaints of the musculoskeletal system in the neck and thoracic spine area and 24% have skin diseases. Most physiotherapists (88%) are satisfied with their work overall. This is aided by a high degree of influence on their work and breaks, by practical application of skills and expert knowledge, high regard for their profession, varied work and a good atmosphere at work. Reservations tend to be about statutory regulations and the social benefits provided by the German healthcare system.

**Conclusion:**

Overall, despite high demands and stress relating to the adequacy of resources, the majority of physiotherapists surveyed seem to be satisfied with their job. The main focus of action to promote the health of physiotherapists should be on preventing skin disease, problems of the musculoskeletal system and accidents caused by stumbles and falls.

## Introduction

Physiotherapists are exposed to diverse demands at work. Awkward postures, when administering treatments such as massages or when transferring immobile patients, can lead to musculoskeletal complaints [1-6]. This impacts on job satisfaction and the willingness to continue in the profession [7]. Until now, few studies in Germany have explored physical stress on physiotherapists. Psychosocial demands can also influence physiotherapists’ job satisfaction. A study by Gröbel [8] examined psychosocial stress factors affecting salaried physiotherapists in the state of Hesse, Germany, and their consequences. It found that the quantitative and emotional demands of work tended to be viewed as moderate and that job satisfaction was rated as high. Until now, no studies have been conducted in Germany on the interaction between physical and psychosocial stress and the job satisfaction of physiotherapists.

This paper analyses routine data on accidents at work and occupational diseases of physiotherapists who work in physiotherapy practices and are insured with the Institute for Statutory Accident Insurance and Prevention in the Health and Welfare Services (BGW). A survey of work-related physical and psychosocial stress and the job satisfaction of physiotherapists was also conducted.

## Material and methods

The Institute for Statutory Accident Insurance and Prevention in the Health and Welfare Services (BGW) routinely records reports of accidents at work and occupational diseases [9,10]. Along with information about the accident and resulting injury, or the type of occupational disease and confirmed cause of illness, the BGW documents the affected person’s activity. It distinguishes between accidents at the workplace or during working hours and accidents on the way to and from work. Accidents that lead to more than three days’ incapacity for work are notifiable. The circumstances that lead to the injury are documented in more detail in a 7% random sample of all notifiable accidents. It is obligatory to report a disease if there are reasonable grounds for suspecting that the disease was caused by exposure at the workplace. For instance, contact with an infectious agent is not notifiable, but an occupationally acquired infection is notifiable [10,11]. Some occupational diseases are subject to a proviso. They can only be recognised if the disease led to relinquishment of the stressful work. This applies mainly to skin diseases and disc-related complaints of the lumbar spine.

In 2011, 136,998 full-time members of the occupational group of physiotherapists were insured with the BGW. The number of full-time workers is calculated on the basis of the number of monthly hours worked by an insured person. A part-time worker who works 80 hours per month is equivalent to 0.5 full-time workers. We analysed accidents at work and en route to/from work, along with occupational diseases, for the years 2007–2011.

In 2012, we also conducted a cross-sectional survey to examine work-related stress and job satisfaction among physiotherapists. In order to obtain a random sample of subjects for this survey, we randomly selected from the BGW dataset of all insured physiotherapists the addresses of 210 salaried, freelance or self-employed physiotherapists working in outpatient practices. After matching the address data, inter alia via the Deutscher Verband für Physiotherapie (ZVK) [12], subjects were sent an anonymous questionnaire plus a separate declaration of consent and a stamped addressed return envelope.

For the survey, we developed a standard questionnaire containing 84 items to be completed by the subjects themselves. In addition to socio-demographic information, there were questions on occupation-specific aspects. In order to quantify physical stress and demands at the workplace, frequent postures, average hand activity and exhaustion at the end of a working day were recorded. In addition, subjects were asked about contact with persons with infectious diseases.

Several questions were taken from the Copenhagen Psychosocial Questionnaire (COPSOQ) and adjusted for the physiotherapists’ field of work. The COPSOQ on psychosocial stress at work was developed at the National Institute for Occupational Health in Copenhagen [13,14] and tested, shortened and standardised for Germany [15,16]. This instrument casts light on demands and satisfaction at the workplace, the working climate and the work-privacy conflict. It has previously been used for several studies of employees in the healthcare sector [17,18].

Another section of the questionnaire is based on the Work Ability Index (WAI) [19]. This instrument is used to ascertain subjects’ ability to work and state of health by recording both their assessment of their own mental and physical fitness for work and currently diagnosed diseases. For the survey of physiotherapists, the range of diseases was reduced and confined to accidental injuries, diseases of the musculoskeletal system, skin diseases, diseases of the vascular system, and mental illnesses.

The *IBM SPSS Statistics 21* program was used to carry out a univariate and bivariate analysis of the data. The study was conducted in accordance with the Federal Data Protection Act. Since it involved the analysis of routine data and an anonymous questionnaire, no ethics committee approval was obtained. The BGW’s data protection officer approved the data protection concept for the study.

## Results

### Occupational diseases and accidents at work

In 2011, the number of insured physiotherapists in outpatient practices was equivalent to 136,998 full-time posts, an increase of 21.5% on the previous year’s 112,718. In the years from 2007 to 2011, physiotherapists reported a total of 25,891 insured events to the BGW. The number of reports in 2011 (n = 6.193) increased by 44.9% compared with 2007 (n = 4.275). The report ratio rose from 17.9 to 22.6 insured events per 1,000 full-time equivalents during this period. Of the insured events reported, 47.8% were notifiable. The most frequent reports concerned accidents at work (n = 14,332, of which 44.3% were notifiable), followed by accidents en route to/from work (n = 8,681, 55.2% notifiable) and occupational diseases (n = 2,878, 42.7% notifiable). The report ratio per 1,000 full-time equivalents increased for all three types of insured event (Table 1).

**Table 1:** **Insurance cases among physiotherapists in the years 2007 to 2011, BGW data**

**Reporting year**	**2007**	**2008**	**2009**	**2010**	**2011**	**Total**
Number of full-time staff	112,718	120,451	126,679	133,134	136,998	--
Total insured events (IE)	4,275	4,673	5,090	5,660	6,193	25,891
IEs per 1,000 full-time staff	17.9	18.1	18.6	20.4	22.6	--
Notifiable IEs	2,017	2,179	2,360	2,717	3,090	12,363
Reported occupational diseases (OD)	458	510	542	600	768	2,878
ODs per 1,000 IEs	4.1	4.2	4.3	4.5	5.6	
Notifiable ODs	189	208	216	263	353	1,229
Proportion of total ODs	41	41	40	44	46	43
Occupational accidents (OA)	2,429	2,645	2,826	3,096	3,336	14,332
OAs per 1,000 IEs	21.5	22.0	22.3	23.3	24.4	
Notifiable OAs	1,058	1,188	1,197	1,369	1,534	6,346
Accidents en route to/from work (AER)	1,388	1,518	1,722	1,964	2,089	8,681
AERs per 1,000 IEs	12.3	12.6	13.6	14.8	15.2	
Notifiable AERs	770	783	947	1,085	1,203	4,788

IE insured event.

OD occupational disease.

OA occupational accident.

AER accident en route to/from work.

The majority of insured events involved women (76.0%). Men were slightly over-represented in accidents at work, accounting for 26.1% of cases – as opposed to 19.4% for occupational diseases and 21.5% for accidents en route to/from work (Table 2).

**Table 2:** **Gender distribution by type of insured event among physiotherapists, BGW data**

	**Occupational disease**	**Occupational accident**	**Accident en route to/from work**	**All**
	**N (%)**	**N (%)**	**N (%)**	**N (%)**
Male	558 (19.4)	3,747 (26.1)	1,869 (21.5)	6,174 (23.8)
Female	2,316 (80.1)	10,563 (73.7)	6,788 (78.2)	19,667 (76.0)
Unknown	4 (0.5)	22 (0.2)	24 (0.3)	50 (0.2)
Total	2,878 (11.1)	14,332 (55.4)	8,681 (33.5)	25,891

Of the 1,229 cases of notifiable occupational disease, an occupational cause was confirmed in 910 cases (74.0%) and an occupational disease was recognised in 57 cases (4.6%) (Table 3). Skin diseases accounted for both the majority of cases reported (n = 897, 73.0%) and for the majority of cases where an occupational cause was confirmed (876, n = 97.7%). Disc-related diseases of the lumbar spine were the second most frequently reported (n = 85, 6.9%) and occupationally caused (n = 16, 1.8%) disease.

**Table 3:** **Type and frequency of notifiable occupational diseases (OD) among physiotherapists for the years 2007 to 2011, BGW data**

**OD number**	**N (%)**	**Occupational cause confirmed (and OD recognised)**
5101 Severe or recurrent skin diseases	897 (73.0)	876 (28)
2108 Vertebral disc-related diseases of the lumbar spine due to many years’ lifting/carrying heavy loads/extreme bending of the torso	85 (6.9)	16 (11)
2101 Tendovaginal conditions or conditions of the tendinous tissue or of the tendonor muscular attachments	49 (4.0)	2 (2)
3101 Infectious diseases	38 (3.1)	11 (11)
2109 Vertebral disc-related diseases of the cervical spine due to many years’ carrying heavy loads on the shoulder	31 (2.5)	0
4301 4302 Obstructive respiratory diseases due to allergenic substances, chemical irritants or substances with a toxic effect	11 (0.9)	5 (5)
Other ODs	118 (9.6)	0
Total	1,229 (100.0)	910 (57)

Of the 11 infections recognised as occupational diseases or irregular health conditions in the period under review, six involved active tuberculosis and one a latent tuberculosis infection (LTBI). There was one case each of hepatitis B and C, and one of an unspecified bacteriological infection (no table).

Around one in five occupational accidents happened en route to or from work and about half of those were road accidents. Of the accidents en route to or from work, 60.8% were road accidents (Table 4). In 68.4% of cases, the type of injury cannot be identified from the accident report. Closed fractures were the most frequent consequence of injury (14.2%). They are somewhat more frequent in accidents en route to or from work than in accidents at work (16.2% and 12.7%) (Table 5). Hands are more often affected by accidents at work than by accidents on the way to or from work (2.8% and 1.3%) (Table 6). However, in both categories of accident, the feet are more frequently affected (4.8% and 3.2%). Polytrauma or head injuries are the consequence of 2.6% of occupational accidents and 3.4% of accidents en route to or from work.

**Table 4:** **Distribution of reported accidents by individual type of accident for the years 2007 to 2011 among physiotherapists, BGW data**

**Type of accident**	**Notifiable**		**Total**
	**Yes**	**No**	
	**N (col%)**	**N (col%)**	**N (col%)**
Occupational accidents*	**6,346 (57.0)**	**7,986 (67.2)**	**14,332 (62.3)**
of which:			
Occupational accidents at work, no road traffic	4,796 (75.6)	6,758 (84.6)	11,554 (80.6)
Occupational accidents on business, not a road traffic accident	703 (11.1)	685 (8.6)	1,388 (9.7)
Workplace accidents on business, in road traffic*	847 (13.3)	543 (6.8)	1,390 (9.7)
**Accidents en route to/from work**	4,788 (43.0)	3,893 (32.8)	8,681 (37.7)
of which:			
Accidents en route to/from work, not a road traffic accident	1,781 (37.2)	1,618 (41.6)	3,399 (39.2)
Accidents en route to/from work that happened in road traffic	3,007 (62.8)	2,275 (58.4)	5,282 (60.8)
Total*	11,134 (48.4)	11,879 (51.6)	23,013 (100)

*row%.

**Table 5:** **Type of injury for notifiable occupational accidents and accidents en route to/from work among physiotherapists, summarised for the years 2007 to 2011, BGW data**

**Type of injury**		**Type of accident**		**Total**
		**Occupational accident**	**Accident en route to/from work**	
Not specified	Number	4,414	3,200	7,614
	%	69.6%	66.8%	68.4%
Closed, fully reversible injury	Number	178	134	312
	%	2.8%	2.8%	2.8%
Closed, bloody injuries with lasting damage to substance	Number	41	44	85
	%	0.6%	0.9%	0.8%
Torsion, sprain	Number	289	329	618
	%	4.6%	6.9%	5.6%
Dislocation	Number	41	30	71
	%	0.6%	0.6%	0.6%
Laceration, cuts	Number	461	176	637
	%	7.3%	3.7%	5.7%
Closed fracture	Number	807	778	1,585
	%	12.7%	16.2%	14.2%
Open fracture	Number	55	50	105
	%	0.9%	1.0%	0.9%
Burning, scalding, chemical burn, radioactive contamination, hypothermia, frostbite, electrical effects	Number	24	0	24
	%	0.4%	0.0%	0.2%
Other injuries	Number	34	41	75
	%	0.5%	0.9%	0.7%
Type of injury unknown or unclassifiable	Number	2	6	8
	%	0.0%	0.1%	0.1%
Total	Number	6,346	4,788	11,134
	%	100.0%	100.0%	100.0%

**Table 6:** **Site of injury for notifiable occupational accidents and accidents en route to/from work among physiotherapists, summarised for the years 2007 to 2011, BGW data**

**Site of injury**		**Type of accident**		**Total**
		**Occupational accident**	**Accident en route to/from work**	
Not specified	Number	4,414	3,200	7,614
	%	69.6%	66.8%	68.4%
Polytrauma, head	Number	167	164	331
	%	2.6%	3.4%	3.0%
Neck, spinal column, arm and leg nerve plexus	Number	206	323	529
	%	3.2%	6.7%	4.8%
Chest, shoulder girdle, back, flank, chest organs	Number	93	107	200
	%	1.5%	2.2%	1.8%
Abdomen, abdominal organs, pelvis	Number	21	32	53
	%	0.3%	0.7%	0.5%
Shoulder, upper arm, elbow	Number	260	179	439
	%	4.1%	3.7%	3.9%
Lower arm, wrist, carpus	Number	286	225	511
	%	4.5%	4.7%	4.6%
Hand	Number	175	64	239
	%	2.8%	1.3%	2.1%
Hip, thigh, patella	Number	75	96	171
	%	1.2%	2.0%	1.5%
Knee joint (excluding patella) calf	Number	345	244	589
	%	5.4%	5.1%	5.3%
Ankle, foot	Number	304	154	458
	%	4.8%	3.2%	4.1%
Total	Number	6,346	4,788	11,134
	%	100.0%	100.0%	100.0%

In the 7% random sample in the years from 2007 to 2011, a total of 303 notifiable occupational accidents and 195 accidents en route to or from work were recorded (Table 7). This is equivalent to 4.8% and 4.1% respectively of all notifiable events and thus somewhat lower than the 7% aimed for.

**Table 7:** **Description of occupational accidents among physiotherapists according to ‘7% statistics’, BGW data**

**Circumstances of accident**	**Occupational accident**		**Accident en route to/from work**	
**Environment where the accident victim was located prior to the accident**	**N**	**%**	**N**	**%**
Physiotherapy practice or healthcare institution	191	63.0	6	3.1
Paths, roads, public domain	76	25.1	187	95.4
Other	36	11.9	3	1.5
**The activity the victim was engaged in immediately before the accident**				
Handling tools	15	5.0	--	
Handling machinery	2	0.7	--	
Driving/handling power-driven conveyances (car etc.)	37	12.2	76	38.8
Driving/handling non-power-driven conveyances (bicycle etc.)	6	2.0	44	22.4
Handling objects	58	19.1	--	
Running, walking, moving	163	53.8	73	37.2
Other	12	4.0	3	1.5
**Event leading to the accident**				
Loss of control of tool, machinery	18	5.9	1	0.5
Loss of control of vehicle or conveyance	36	11.9	111	56.6
Slipping, stumbling, falling	130	42.9	67	34.1
Uncoordinated, inappropriate, unsuitable movements	76	25.1	5	2.6
Lifting and carrying objects	10	3.3	--	
Surprise, fright, violence	7	2.3	5	2.6
Other	26	8.6	7	3.6
**Contact via which the victim was injured**				
Contact with electrical current, temperatures, hazardous substances	7	2.3	1	0.5
Colliding with or running into a stationary object (the victim moves)	113	37.3	104	53.1
Contact with a sharp, pointed object	15	5.0	1	0.5
Hit by collision with a moving object	54	17.8	70	35.7
Contact with a hard or rough object	10	3.3	1	0.5
Jammed, squashed	11	3.6	2	1.0
Acute excessive physical strain	79	26.1	14	7.1
Acute excessive mental strain	1	0.3	--	
Blow, kick, push, bite	7	2.3	3	1.5
Other	5	1.7	--	
**Most important object connected with the accident**				
Building, building installations	139	45.9	80	40.8
Tools, machinery	16	5.3	--	
Vehicles	43	14.2	103	52.6
Office furnishings, personal equipment, kitchen appliances	50	16.5	--	
Humans and other living beings	40	13.2	7	3.6
Other	15	5.0	6	3.1
Total	303	100.0	196	100.0

As expected, the majority of occupational accidents occurred in physiotherapy practices. However, in line with the high proportion of accidents en route to or from work, 25.1% of accidents took place on paths and roads. In the case of accidents en route to or from work, paths and roads were the predominant site of the accident (95.4%). Running, walking or moving were the most frequent activities engaged in immediately before the accident, both in the case of occupational accidents (53.8%) and in the case of accidents en route to or from work (37.2%) (Table 7). Driving cars or other motor vehicles (38.8%) and the use of a bicycle or similar means of transport (22.4%) were further frequent activities in the run-up to an accident en route to or from work. These activities played a subordinate role in the case of occupational accidents, at 12.2% and 2% respectively.

In occupational accidents, slipping, stumbling or falling (42.9%) and uncoordinated, awkward movements (25.1%) were the most frequent events leading to an accident. With accidents en route to or from work, loss of control over a vehicle or means of transport (56.6%), along with slipping, stumbling or falling (34.1%) were the most frequent events leading to an accident. In keeping with these accident-triggering events, colliding with or running up against an object (37.3% and 53.1%) and being hit by a moving object (17.8% and 35.7%) were the most frequent types of contact leading to an accident. Contact with sharp, pointed objects was rarely a reason for injury (5% and 0.5%). Blows, kicks, pushes or bites were rare occurrences both in occupational accidents (2.3%) and in the case of accidents on the way to or from work (1.5%). Tools and machinery were involved in only 5.3% of occupational accidents. In the case of accidents en route to or from work, as expected, vehicles played a predominant role (52.6%). Human beings or other living things played a role in 13.2% of occupational accidents and 3.2% of accidents en route to or from work. However, only a small proportion of these accidents (7 out of 40 occupational accidents and 3 out of 7 accidents on the way to or from work) were triggered by violence (blow, kick, push, bite) (Table 7). In three out of a total of ten such cases, animals rather than human beings were the cause of the accident (no table).

### Survey of physiotherapists

Eighty-five physiotherapists took part in the survey, representing a response rate of 41%. Twice as many women as men participated in the survey. The majority were in the 40 to 49 and 50 to 59 age groups, while there were only a few representatives of the under-30 age group. Sixty-seven per cent of the subjects had been working in physiotherapy for more than 20 years and 84% were freelance or self-employed proprietors of their own practice. In response to the question about their main activities, as expected 95% of respondents stated that they frequently administered physical therapy, while 87% engaged in mobilisation and manual therapy and 46% performed lymphatic drainage. Less frequently, they mentioned electrotherapy, patient transfer or hydrotherapy. Additional activities stated primarily included massages. Table 8 contains further information about the study population.

**Table 8:** **Description of study population as regards job satisfaction of and stress on physiotherapists, 2012**

**Study population**	**N**	**%**
Total	85	100.0
**Gender distribution**		
Male	28	32.9
Female	57	67.1
**Age**		
Under 30 years	4	4.7
30–49 years	15	17.6
40–49 years	28	32.9
50–59 years	27	31.8
Over 59 years	11	12.9
**Professional experience in physiotherapy**		
0–10 years	12	14.1
11–20 years	16	18.8
Longer than 20 years	57	67.1
**Working hours**		
Full-time (≥35 hours per week)	68	80.0
Part-time (<35 hours per week)	17	20.0
**Overtime in the last twelve months**		
Every day	11	12.9
Several times a week	29	34.1
Several times a month	14	16.5
Several times a year	20	23.5
Never	11	12.9
**Professional status**		
Self-employed	71	83.5
Employee	14	16.5
**Home visits**		
Frequent	70	82.4
Seldom or never	15	17.6
**Tasks mainly performed**		
Physical therapy	81	95.3
Mobilisation/manual therapy	74	87.1
Manual lymphatic drainage	39	45.9
Electrotherapy	16	18.8
Patient transfers	14	16.5
Hydrotherapy	4	4.7

#### Work demands and stress

In connection with quantitative demands at work, one in four physiotherapists said they often had to work very fast and more than half said their work mounted up. In addition, over 50% of respondents were subject to emotional stress (Table 9). One in three physiotherapists found the time spent at work to be a hindrance to their own private responsibilities. However, only 15.3% of respondents said that their work as a physiotherapist interfered with their private and family life.

**Table 9:** **Work stress among physiotherapists (N = 85)**

**Stressor**	**N**	**%**
**Working fast**		
Frequently	22	25.9
Seldom	63	24.1
**Work is unevenly distributed and mounts up**		
Frequently	53	62.4
Seldom	32	37.6
**Not enough time for all tasks**		
Frequently	50	58.8
Seldom	35	41.2
**Work involves emotionally stressful situations**		
Frequently	50	58.8
Seldom	35	41.2
**Work–privacy conflict**		
Work interferes with private and family life		
Yes	13	15.3
No or undecided	72	84.7
**Time spent working hinders private responsibilities**		
Yes	27	31.8
No or undecided	57	68.2

As regards physical demands at work, the physiotherapists were asked to state the postures they frequently adopted while working. It seems that they often had to adopt a torso posture between 45° and 90° when working with patients. However, they rarely bent their torso to an angle of more than 90°. Only 6% treated patients in a squatting position. Physiotherapists work a lot with their hands, but even though the estimated effort required for average hand activity is high, at 84% (Table 10), 79% of respondents stated that they made regular hand movements without a break.

**Table 10:** **Physical demands of the typical physical work performed by physiotherapists surveyed 2012**

**Physical demands of typical daily work**	**N**	**%**
Torso inclination of 45° to 90°	47	55.3
Squatting posture	16	18.8
Torso inclination >90°	15	17.6
Kneeling posture	15	17.6
Hands above shoulder level	13	15.3
Squatting	5	5.9
**Hand activity**		
No regular hand activities	1	1.2
Slow movements with breaks	12	14.1
Regular movements without breaks	67	78.8
Fast movements without breaks	5	5.9
**Effort required, hands**		
Moderate	14	16.5
High	71	83.5

In the case of treatment of patients with infectious diseases, the highest proportion (33%) mentioned contact with patients with multi-resistant pathogens. The next highest rate of contact was with patients with hepatitis (27%) and HIV (15%). Only a few physiotherapists came into contact with childhood diseases (6%) such as whooping cough or measles or tuberculosis (2%), while 41% of respondents stated that they had no contact with patients with infectious diseases (no table).

#### Job satisfaction

With regard to their mental power reserves, 81% of the physiotherapists questioned stated that they had enjoyed their work of late. Nearly all participants in this study (92.9%) had a major influence on their own work, saw it as important and meaningful (97.6%) and could decide for themselves when to take a break (76.5%). They described their work as varied and felt motivated and engaged (90.6%). Moreover, they were able to apply their learned skills and expert knowledge. In addition, 80% said that the working atmosphere among colleagues was good and nearly half of respondents said they exchanged views and information at a professional level. In contrast, there was hardly any professional interchange with medical practitioners (8%) (Table 11).

**Table 11:** **Description of resources that positively reinforced the work of physiotherapists in 2012**

**Resources**	**N**	**%**
High job satisfaction	68	80.0
Daily tasks performed with pleasure	69	81.2
Influence on own work activity	79	92.9
Self-determined break times	65	76.5
Views own work as important and meaningful	83	97.6
Work is varied	74	87.1
Motivated and engaged in work	77	90.6
Application of acquired skills and expert knowledge at work	79	92.9
Good working atmosphere with colleagues	68	80.0
Frequent professional interchange with colleagues	34	40.0
Frequent professional interchange with doctors	7	8.2

An open question was used to record which factors would further enhance the physiotherapists’ job satisfaction. Around one third mentioned better pay. Some also wanted more time for patients and less bureaucracy, especially when working with health insurers and in respect of the current government directive on prescriptions for therapeutic remedies (Heilmittelrichtlinie). Also mentioned were better cooperation with doctors and a generally higher level of recognition of their profession (no table). Overall, 80% of respondents were satisfied with their work (Table 11).

#### Complaints and diseases

Half of the respondents mentioned a disease of the cervical and thoracic spine, 36.5% a disease of the lumbar spine and 30.6% a disease of the iliosacral joint (Table 6). Around half the physiotherapists (47%) diagnosed these diseases themselves (no table). Twenty-seven per cent of the physiotherapists said they had wrist problems (carpal tunnel syndrome, rhizarthrosis), of which 60% were self-diagnosed. Knee joint complaints (20%) were somewhat less frequent (Table 12).

**Table 12:** **Information on diseases that may be attributable to the profession of physiotherapist 2012**

**Diseases of the musculoskeletal system**	**N**	**%**
Lumbar spine and thoracic spine	43	50.6
Lumbar spine	31	36.5
Iliosacral joint	26	30.6
Wrist	23	27.1
Thumb-saddle joint	23	27.1
Knee joint	17	20.0
**Skin diseases**	20	23.5
**Minor mental illnesses**	21	24.7
**Unfit for work in the last twelve months**		
No	53	62.4
Up to one month	29	34.1
Longer than one month	3	3.5

Skin diseases (skin irritation, allergic skin rash/eczema, fungal infections) had been diagnosed in one quarter of respondents. Likewise, 25% of the physiotherapists surveyed suffered slight mental problems such as mild depression, anxiety, tenseness, insomnia and burnout (Table 6).

The majority (80%) of physiotherapists assessed their current physical (74%) and mental (73%) work capacity as good compared with when they were at their best. At the time of the survey, 46% had neither a disease nor an injury that impeded their work. However, 39% said they had problems in doing their work and 27% said that as a result they were sometimes forced to work more slowly or to change their working methods.

Of the respondents, 61% said they often felt exhausted at the end of a working day. Approximately one third had often considered leaving their profession during the previous twelve months, with 55% citing physical problems as the main reason for leaving. This was followed by poor pay (32%) and mental stress (24%). However, 59% had never considered leaving the profession. Only 4% of the physiotherapists said they were unfit for work at the time of the survey (no table). When asked about sickness in the past year, 53 (62.4%) said they had not been ill, while 34.1% had been unfit for work for up to one month and 3.5% for longer than one month (Table 12). Eighty percent of respondents said they were fairly certain that they would be able to do their work without problems in two years’ time (no table).

## Discussion

According to our survey, work in a forward-bending posture and repetitive hand movements involving physical effort represent typical strains on physiotherapists. Back problems and hand and finger problems are correspondingly frequent. However, the design of the study as a cross-sectional study and the relatively small sample size make it impossible to analyse causal interrelations. Despite the high quantitative work demands, physiotherapists were satisfied with their job.

### Complaints and diseases

The data provided by the Institute for Statutory Accident Insurance and Prevention in the Health and Welfare Services (BGW) on occupational diseases showed skin diseases (73%) to be the most frequently reported occupational disease. However, in the context of our cross-sectional study, skin diseases played a subordinate role in contrast to diseases of the musculoskeletal system. A study by Cromie [20] reported that physiotherapists who often performed hydrotherapy with their patients had a higher probability of suffering skin problems. In this case, 37% of the total suffered from skin infections. In the random sample in our study, only a few therapists treated patients with hydrotherapy. Even so, a total of 24% suffered from irritated skin, allergic rashes or eczemas and fungal infections. From this, one can deduce that along with hydrotherapy, other factors such as increased use of disinfectants in hand hygiene might be responsible.

One of the professional risks run by physiotherapists is that of contact with patients with infectious diseases. In Cromie’s study [20], 13% of respondents said they had caught an infectious disease through their work as a physiotherapist. According to our analysis of the BGW data, infectious diseases played only a subordinate role as an occupational disease (3% of all reports). In the five-year survey period, 38 infections were reported and 11 were recognised as an occupational disease. Tuberculosis accounted for the highest proportion (7 out of 11). Physiotherapists who practise breathing therapies run a special risk of TB infection [11,21,22]. One case each of hepatitis B and hepatitis C virus infections were recognised as occupational diseases. Thus, blood-borne virus infections seem to be rare among physiotherapists. This is in line with the generally positive trend in blood-borne virus infections among healthcare professionals in general in Germany form whom the number of claims concerning blood-burne virus infections decreased from above 500 in the year 1999 to below 200 in the year 2009 [10]. In the cross-sectional study described here, it appears that frequent contact with patients with multi-resistant pathogens (33%) and hepatitis in physiotherapy calls for efficient hygiene measures. Matching against the infectious diseases reported indicates that hygiene measures are effective.

Physiotherapists are exposed to physical strains in their profession. The BGW data shows that 7% of the notifiable occupational diseases involved ‘vertebral disc-related diseases of the lumbar spine due to many years’ lifting or carrying heavy loads or extreme bending of the torso’, while 2.5% reported a ‘vertebral disc-related disease of the cervical spine due to many years’ carrying heavy loads on the shoulder’. Admittedly, none of the cervical spine diseases reported was recognised as an occupational disease because the physiotherapists did not fulfil the work-related prerequisites for recognition. Studies by Bork, 1996 [2], Cromie, 2000 [23], Campo, 2008 [3] and Grooten, 2011 [5], also ascertained that musculoskeletal diseases in physiotherapists occurred most frequently in the lower back (lumbar spine, iliosacral). Our study, too, showed that along with a higher occurrence in the area of the cervical and thoracic spine (51%), the physiotherapists also suffered musculoskeletal diseases of the lumbar spine and iliosacral complaints, each at a frequency of around 30%. As confirmed by other studies [5,6], these appear to have been provoked by awkward body postures: 55% of the physiotherapists surveyed stated that they often had to work in a forward-bending position with their torso at an angle of 45° to 90°, which can lead to musculoskeletal problems in the spinal column area. In an occupational health study to assess strain on the lumbar spine, it was found that vertebral disc L5/S1 is subject to the most strain when lifting heavy objects and when the torso is bent at an angle of 50°. In addition, strong compressive forces impact on the disc when the torso is in a static forward-bending position at an angle of 90° [24].

As regards patient transfers, 17% of respondents in our study said that they mainly did this work themselves. Along with awkward work postures, this factor is seen as one of the most problematic as far as the development of disc-related complaints and diseases is concerned [2,6]. This problem primarily affects physiotherapists (more than 80% of those in our investigation) who make frequent home visits where they find conditions that impede optimal patient care [25].

Frequent home visits involving journeys in road traffic may also be a reason for the high number (2,778) of accidents en route to/from work reported to the BGW.

Physiotherapists often work with their hands (massages, mobilising, manual therapy), which places a great strain on the hand and thumb joints [2,5,23]. This can be a risk factor for ‘tendovaginal conditions or conditions of the tendinous tissue or of the tendonor muscular attachments’ that affected 4% of the physiotherapists with notifiable occupational diseases (Table 3). Almost half the therapists who worked only with their hands suffered from carpal tunnel syndrome and had to take protective and corrective measures (changing their work posture, wearing wrist braces, taking painkillers, undergoing surgery) in order to continue working in their profession [1]. Physiotherapists who suffered from diseases of the thumb-saddle joint also had to adapt their way of working to cope with the complaint [26]. A major Australian study found that mainly therapists who administered massages and manual therapy suffered from thumb-saddle joint problems. It also found increased instability in the thumb joints [27].

The majority of respondents made regular hand movements involving a great deal of hand activity without a break. As a result, more than a quarter of respondents suffered from musculoskeletal complaints of the hand or thumb-saddle joints such as carpal tunnel syndrome or rhizarthrosis. However, this study found no significant connection between frequent manual treatment (mobilisation, manual therapy) and diseases of the hand joint (no table). Nonetheless, it seems reasonable to analyse the causes of musculoskeletal complaints in physiotherapists in a more differentiated way, as is already happening in an ongoing study [28].

Some physiotherapists seemed to think that the fact that they know how work-related musculoskeletal diseases could be prevented protected them against such diseases and that they as role models ought not to suffer from any such diseases [4,29]. Others preferred to diagnose and treat themselves and had not consulted a doctor. This is attributable to the skills in this profession. Campo’s 2008 study [3] showed that only 13% of all physiotherapists had consulted their doctor in a one-year period, but that more than 50% suffered from work-related musculoskeletal complaints. Our survey, too, found that roughly half of all diseases of the musculoskeletal system were self-diagnosed. Nonetheless, a visit to a doctor and further diagnosis could potentially further isolate the cause of existing symptoms and more systematic treatment might bring results more quickly or prevent illness. It might also encourage the individual’s admission of illness and increase social support [4].

### Work stress

Almost 40% of the physiotherapists in this study said they could do their work, but suffered from complaints, while others were forced to work more slowly or to change their working methods. Almost two thirds felt exhausted at the end of a working day. Cromie found that 17% of physiotherapists changed their specialism or even their profession on account of musculoskeletal diseases [23]. In our study, 55% said that they were most likely to leave their profession because of physical problems.

Despite this stress, 46% of the physiotherapists surveyed did not feel that their work was impaired. Nearly two thirds had not been ill in the previous year. Three quarters assessed their current fitness for work and its physical demands as good and were fairly certain that they would still be able to practise their profession in two years’ time without any problems. This cannot be confirmed on the basis of the literature. In Campo’s phenomenological study, most physiotherapists were worried about the future [29]. Among the physiotherapists we surveyed, work-related physical stress appeared to lead to diseases and dissatisfaction only to a limited extent.

Stress at the workplace can impact heavily on working people’s psyches and satisfaction with life [30] and can lead to work-related musculoskeletal diseases or to a change of workplace [3,7]. In a 2010 study by Santos, 36% of subjects said they felt stressed. Our study found that 24% cited mental stress as the crucial reason for a possible change of profession. Stressors include insufficient independence and a lack of recognition by society in general and in the healthcare sector in particular [31]. The physiotherapists in this study would appreciate greater recognition from doctors, health insurers and society at large.

Stress can also impact on the psyche. The majority of physiotherapists assessed their current fitness for work as regards mental demands as good, one quarter as mediocre and only two subjects as poor. A quarter of those surveyed suffered from minor mental complaints. A study of healthcare workers in Cyprus [32] showed that 46% found their profession stressful and 21% were suffering from burnout.

For the physiotherapists in our study, mental stress seemed to play a subordinate role. It did not seem to impact negatively on their job satisfaction. The conflict between work and family can lead to social stress. Burtchen found in 2006 that more than 35% of the physiotherapists she surveyed cited stress in their private lives as a stress factor at work, but also that stress at the workplace was often relieved in private life [33]. Our study findings tally with those of Gröbel [8], namely that professional demands on physiotherapists tend to have only a minor effect on their private and family lives.

### Job satisfaction

The results of this study show that nearly 90% of the physiotherapists surveyed were satisfied with their jobs on the whole. This is a very high percentage, but tallies with the findings of other studies that also found a high level of job satisfaction among physiotherapists [34-37]. Karasek’s job-demand control model [38] says that high demands with low control may lead to health and mental deficits. At the same time, high demands accompanied by high influencing possibilities and control over the work performed minimise health stress and can improve regenerative capacities (active workers). Although high demands ensure a certain stress level, they also increase motivation [39,40]. Earlier studies [7] had already shown that physiotherapists tend to belong to the group of active workers. This is an important factor for a high level of job satisfaction.

The demands on physiotherapists are having to work quickly, unevenly distributed work that mounts up because they sometimes have less than 15 minutes per patient and too little time to complete all their tasks, which include documentation. Roughly a quarter said that having more time for patients, documentation and home visits, along with a reduction in bureaucracy, would lead to increased job satisfaction. In addition, there are emotionally stressful situations such as patients’ sad stories or the working atmosphere. The physiotherapists in this study exerted a high level of control primarily because the majority of those surveyed worked independently. So in an ideal case, the therapists could decide for themselves how a treatment should proceed or when to plan breaks. Unfortunately, this is not always easy in practice because statutory regulations in particular place constraints on the welfare system. According to the duty to check, as laid down in the current 1 July 2011 directive on prescriptions for therapeutic remedies (Heilmittelrichtlinie), physiotherapists may only provide and charge for their services if there are no formal errors in the prescriptions issued by doctors [41,16]. As a result, these prescriptions must be checked for accuracy each time before treating a patient, which further reduces the little time available to therapists. Given the difficulties of working with some health insurers, a quarter of the therapists surveyed cited this as a negative factor impacting on job satisfaction. Moreover, collaboration with some medical practitioners did not always work. Only 8% of the physiotherapists surveyed said that there was sufficient communication and professional interchange with doctors. Already in Bergmann’s study, 55% said that doctors expected them to act independently [42]. In Burtchen’s study, too, they complained about insufficient cooperation with doctors [33].

Success is another important factor in physiotherapists’ job satisfaction. In a phenomenological study by Takeuchi, the success of physiotherapists is described primarily as a circular process that emerges during the course of their career and is developed on the basis of positive results in terms of patients, their career and reputation [43]. An employee’s salary can also be seen as success at work: roughly one third of those surveyed for this study were dissatisfied with their salary and wanted better pay. This can lead to job dissatisfaction [44].

In order to deal with and compensate for the physical and psychosocial demands and strains, resources are needed to prevent mental stress reactions [35]. Physiotherapists employed in larger rehabilitation centres or hospitals often complain that they have too little scope and work under too much time pressure [8]. Since most of the subjects in the present study worked independently as practice proprietors or freelancers, the majority of them had more scope to arrange their working hours and breaks, and more influence on their own work. Varied work with different patients, the application of learned skills and expert knowledge and the feeling that the work being done is important and meaningful reinforce job satisfaction. Thus, nearly all of those questioned felt motivated and engaged, a feeling that was reinforced by good social relationships. A relaxed and collegial work climate makes a substantial contribution toward employees’ health. If relationships between people at the workplace do not function, this leads to dissatisfaction [44,45]. Eighty per cent of the physiotherapists surveyed, however, described the atmosphere among colleagues at the workplace as pleasant and felt that they were part of a community. Professional interchange with colleagues about difficult cases or new techniques also helps in solving stressful problems and enhances one’s self-esteem in respect of one’s own professional competence.

### Limitations

The response rate for this study was relatively low at 41%, as only 85 physiotherapists took part in the survey. Differing response behaviour may lead to selection bias. Physiotherapists who were fairly satisfied with their profession and therefore, with appropriate time management, had enough time to complete the questionnaire may have been the main participants in this study and may therefore have distorted the response behaviour positively. On the other hand, those who were very dissatisfied with their profession may also have taken the opportunity to express their discontent. These response behaviours may balance each other out and deliver a fairly accurate image of the reality. Since we only wrote to practices, and in the main independent physiotherapists participated in this study, the findings can hardly be transferred to employees in practices or hospitals.

Problems that were not addressed in our questionnaire but which, according to the results of the open question about other factors, have a major influence on the job satisfaction of physiotherapists, are statutory regulations due to the current catalogue of remedies as well as working with health insurers. These aspects should be included in further studies.

## Conclusion

Despite the resource-related physical and psychosocial stress, there is a high rate of job satisfaction among physiotherapists. Limitations tend to be seen in statutory regulations and welfare benefits of the German healthcare system. In order to minimise the stress on physiotherapists, preventive measures are still required, especially in order to prevent awkward postures and infections. In addition, some changes in the regulations governing the healthcare system could ensure less bureaucracy and greater concentration on patients. Attention should be paid to sufficient protection for physiotherapists’ resources so that this profession remains as satisfied as it is now.
